# MiRNA-Responsive CRISPR-Cas System via a DNA Regulator

**DOI:** 10.3390/bios13110975

**Published:** 2023-11-07

**Authors:** Dayoung Yun, Cheulhee Jung

**Affiliations:** Department of Biotechnology, College of Life Sciences and Biotechnology, Korea University, Seoul 02841, Republic of Korea; ekdud523@korea.ac.kr

**Keywords:** CRISPR-Cas9, DNA regulator, miRNA, specific regulation

## Abstract

Clustered regularly interspaced short palindromic repeats (CRISPR)- CRISPR-associated protein 9 (Cas9) genome editing technology is widely used for gene editing because it provides versatility in genetic manipulation. Several methods for regulating CRISPR activity already exist for accurate editing, but these require complex engineering. Thus, a simple and convenient regulatory system is required. In this study, we devised a CRISPR activation system using a DNA regulator that can be activated by miRNAs. The designed regulator was divided into two parts. The inhibition component consisted of the protospacer-adjacent motif (PAM) and seed sequence, which are important for Cas9 target recognition and bind to the ribonucleoprotein (RNP) complex for inhibition. The miRNA recognition component has a single-stranded toehold DNA for target miRNA binding and a partial double-stranded DNA complementary to the remaining miRNA sequence. In the presence of target miRNAs, the structure of the regulator is disrupted by the miRNAs, leading to its dissociation from the RNP complex and subsequent restoration of CRISPR activity. This method is easy to design and can be applied to various miRNAs via simple sequence manipulation. Therefore, this strategy provides a general platform for controlled genome editing.

## 1. Introduction

The clustered regularly interspaced short palindromic repeat (CRISPR)-CRISPR-associated protein (Cas) system derived from the bacterial immune system is widely used as an effective genome editing tool in various fields [1,2,3]. The most studied Type II Cas9 system comprises guide RNA (gRNA) and Cas9 proteins [4]. Cas9 is an RNA-guided endonuclease that forms a ribonucleoprotein (RNP) complex with gRNA to induce double-strand breaks (DSBs) in target DNA. Unlike other gene editing technologies such as ZFN and TALEN, the CRISPR system can be easily applied to various targets by changing only the spacer sequence of the gRNA. Further, various Cas9 variants such as dCas9, base editors, and prime editors have contributed to enhancing the scope and precision of genome editing [5]. These characteristics have facilitated the utilization of the CRISPR system across a broad spectrum of applications ranging from genetic editing to biosensing [6,7].

In particular, the RNP form of CRISPR is preferred because of its lower off-target effects and higher efficiency compared to those of other forms, such as plasmids and mRNA, leading to the development of various regulatory strategies for utilizing this form. Currently, the methods for regulating RNP activity involve making them responsive to small molecules, miRNAs, or light [8,9,10,11,12,13,14,15,16,17,18]. However, small molecules, like doxycycline, have potential cellular toxicity, and light-based methods require additional equipment, which are limitations [19,20].

miRNAs have the advantage of being endogenously expressed in cells, which results in low toxicity and eliminates the need for exogenous introduction. Additionally, miRNAs are small, non-coding RNA molecules that play a crucial role in the post-transcriptional regulation of genes. They are particularly recognized for their potential as biomarkers for various diseases, such as cancer and cardiovascular disorders [21,22,23,24]. Therefore, developing methods for the sensitive and specific detection of miRNAs is of paramount importance in the field of molecular diagnostics.

**Figure 1:**
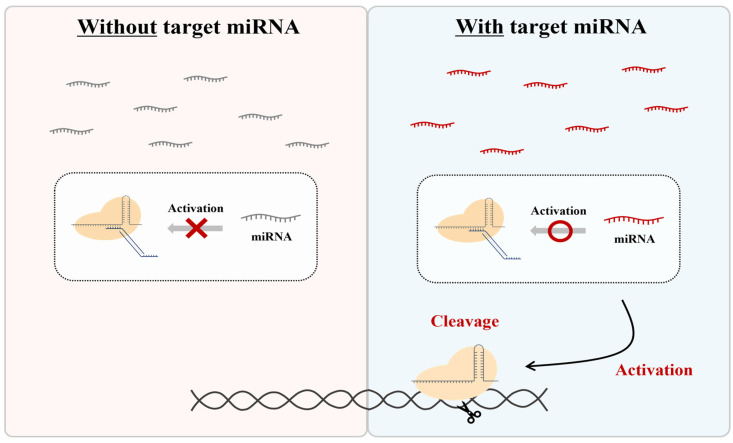
Scheme of CRISPR regulation by miRNA-responsive DNA regulators. This system is only activated in the presence of a specific miRNA.

Therefore, in this study, we aimed to develop a system that distinguishes itself from other regulators by using nontoxic, endogenously expressed miRNAs. To achieve this, we created a method to specifically control the activity of the CRISPR-Cas9 RNP form using an independent ‘DNA regulator’ responsive to miRNA. While the CRISPR-Cas9 system is not inherently capable of recognizing RNA sequences, we introduced a unique DNA regulator that is responsive to specific miRNAs, allowing for the modulation of CRISPR activity (Figure 1). This approach uses the RNP form and naturally expressed miRNA as activators, employing an additional DNA regulator without any component modification. This enables the CRISPR system to be activated by target miRNAs, allowing the system to cut specific DNA sequences and combine them with various amplification and detection platforms.

This system offers a straightforward and versatile design for the CRISPR system, providing a promising tool for applications in therapeutics and biosensing by allowing the CRISPR system to operate in response to target miRNAs, which in turn cleave specific DNA sequences, thereby combining with different amplification and detection platforms.

## 2. Materials and Methods

### 2.1. sgRNA and Primer Design

Single guide RNAs (sgRNA) were designed using CHOPCHOP. The sgRNAs were purchased from Synthego (Redwood City, CA, USA). Primers were designed using the IDT primer design tool and the NCBI Primer BLAST. The designed primers were purchased from IDT.

### 2.2. DNA Substrate Construction

Genomic DNA (gDNA) was isolated using the G-spin Total DNA Extraction Kit (Intron Biotechnology, Gyeonggi-do, Korea, cat# 170046). The target region was amplified from 150 ng of gDNA using Q5 High Fidelity DNA polymerase (NEB, Hitchin, UK, cat# M0491L) and primers. The PCR program was as follows: 98 °C for 30 s; 30 cycles of 98 °C for 10 s, 65 °C for 20 s, 72 °C for 20 s; and 72 °C for 2 min. Finally, the PCR product was purified using the Monarch PCR&DNA clean-up kit (NEB, cat# T1030S).

### 2.3. In Vitro Inhibition Assay

For the test, 1 µM spCas9 nuclease (IDT, Coralville, IA, USA, cat# 1081059) and 1 µM sgRNA were preincubated in reaction buffer (10 mM HEPES, 100 mM NaCl, 5 mM MgCl_2_, and 0.1 mM EDTA). The formed 100 nM RNP was mixed with a 100 nM DNA regulator and incubated for 10 min at room temperature. Next, 5 nM target DNA amplicon was added to the mixture and incubated for 1 h at 37 °C. The reaction was then stopped with a Proteinase K (VIAGEN biotech, Los Angeles, CA, USA), followed by incubation at 56 °C for 10 min. The final product was analyzed using 1.5% TBE agarose gel electrophoresis.

### 2.4. In Vitro Reactivation Assay

Similar to the in vitro inhibition assay, we created a DNA regulator–RNP complex. To verify the reactivation using miRNA, it was added to the DNA regulator-RNP complex at a 1:2 molar ratio (regulator-RNP complex: miRNA or mRNA). After incubation for 10 min at room temperature, the DNA amplicon was added and incubated for 1 h at 37 °C. The reaction was stopped by incubation at 56 °C for 10 min with Proteinase K (VIAGEN biotech). The final product was analyzed using 1.5% TBE agarose gel electrophoresis.

### 2.5. EMSA

For the electromobility shift assay (EMSA), Cas9 nuclease and sgRNA were preincubated at equal molar ratios in reaction buffer (10 mM HEPES, 100 mM NaCl, 5 mM MgCl_2_, and 0.1 mM EDTA). The formed 100 nM RNP was mixed with a DNA regulator at a molar ratio of 1:1 and then incubated for 10 min at room temperature. The mixture was then incubated at 37 °C under the same conditions as those used for the in vitro cleavage assay. The mixtures were run on 7.5% native polyacrylamide gel (30:1 acrylamide/bisacrylamide) in 1× TBE (Tris-borate EDTA electrophoresis buffer). After electrophoresis, the gels were visualized using SYBR Gold gel staining.

## 3. Results

### 3.1. Design of DNA Regulator

**Figure 2:**
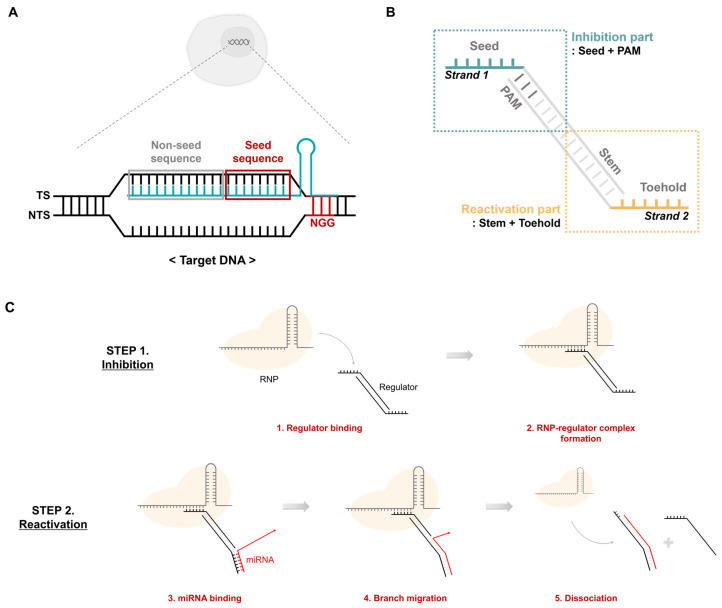
CRISPR activity regulation using a DNA regulator. (**A**) Features of the target dsDNA. The target DNA comprises a target strand and a non-target strand. The target strand contains the target sequence complementary to sgRNA. The non-target strand has an NGG PAM sequence. TS: target strand, NTS: non-target strand. (**B**) Structure of the DNA regulator. It has two separate stands. Strand 1 contains the PAM and seed sequence, and strand 2 includes the toehold and stem complementary to the specific miRNA. (**C**) Mechanism of CRISPR-cas9 regulation by the DNA regulator. Step 2 only occurs under the presence of abundant target miRNA conditions.

In the CRISPR-Cas9 system, target DNA cleavage proceeds through three main steps. The first step involves binding of Cas9 RNP to the target DNA through its RNA-guided mechanism. The second step is the formation of the RNA-DNA heteroduplex, and the last step is the cleavage of the target DNA by Cas9 nuclease activity. In the second step, the target DNA is divided into a target strand (TS) that binds to the gRNA and a non-target strand (NTS) that does not bind (Figure 2A). Particularly, the TS is divided into seed and non-seed sequences. Together with the protospacer-adjacent motif (PAM) sequence, the seed sequence plays an important role in the recognition and binding of RNP to the target DNA. Based on these features, previous studies have addressed aptamers that include PAM and some seeds to inhibit the activity of the CRISPR-Cas system [25]. This only inhibits activity by binding to RNP. However, in this study, an additional module that can respond to miRNA was introduced. Thus, we designed a DNA regulator that ultimately controls RNP activity in response to specific miRNAs (Figure 2B). Strand 1, which mimics the TS, has seed, PAM, and stem sequences that can bind to strand 2. Strand 2 contains an NGG PAM sequence, a toehold, and a stem sequence that can bind to miRNA. The DNA regulators formed from strands 1 and 2 are divided into inhibition and reactivation parts depending on their roles. The inhibition part includes the seed and PAM sequences and suppresses the activity of the CRISPR-Cas system by being recognized as the actual target. The reactivation component responds to the target miRNA, thereby reactivating the RNP in suppressed states.

Regulation of CRISPR-Cas system activity using a DNA regulator is shown in Figure 2C. Similar to the target, the DNA regulator binds to the RNP to form an RNP-regulator complex that maintains an inhibited state even in the presence of the target DNA (Figure 2C, Step 1). However, reactivation is only possible when a specific miRNA exists through the toehold and stem sequences on strand 2. First, the miRNA and toehold form a complementary bond, followed by sequential branch migration along the stem sequence. Thus, the regulator separates from the RNP, freeing the RNP complex and allowing it to recognize and cleave the actual target (Figure 2C, Step 2).

### 3.2. Inhibition of CRISPR-Cas9 Activity with DNA Regulator

**Figure 3:**
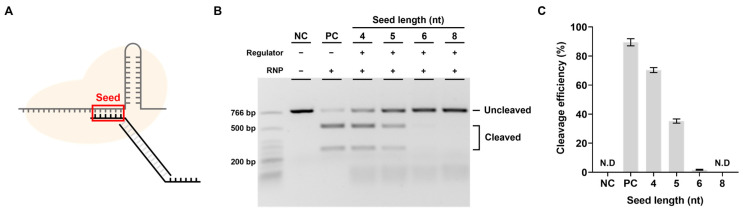
Inhibition efficiency relative to seed length. (**A**) The most important part for inhibiting the cleavage activity of RNP. (**B**) Results of in vitro cleavage analysis on the inhibition efficiency of the designed regulator. The RNP: Regulator molar ratio is 1:1. NC: negative control, PC: positive control. (**C**) Quantification of the ratio of cleaved target DNA from. ND: not detected (**B**). Error bars represent the mean ± SD from three independent experiments.

To determine whether a DNA regulator can control CRISPR-Cas9 activity, we first verified the efficiency of the DNA regulator in inhibiting CRISPR-Cas9 cleavage activity in the absence of miRNA. An important part of the inhibition efficiency was thought to be the seed sequence length, which could affect binding stability between the DNA regulator and spacer sequence (Figure 3A). Therefore, the inhibition effect was expected to increase as the seed sequence length increased, and the goal was to determine the length corresponding to the maximum inhibition efficiency.

We varied the length of the seed sequence in the DNA regulator and conducted experiments targeting EMX1. The inhibitory effect of the regulator was confirmed by the target DNA cleavage efficiency in an in vitro cleavage assay (Figure 3B,C). As expected, the longer the seed sequence, the lower the target cleavage efficiency; when the seed sequence was 6 nt or longer, activity was effectively inhibited. To determine whether this inhibitory effect was due to the DNA regulator, with a seed and PAM sequence recognized as the target, we measured the RNP cleavage efficiency when only strand 1, which could inhibit activity by complementary binding to the spacer, was present (Supplementary Figure S1). The results showed a similar cutting efficiency to that of RNP in the absence of a DNA regulator when checked using the same method. This shows that RNP activity cannot be suppressed by single-stranded DNA containing the seed sequence alone and that the PAM sequence is essential for the DNA regulator to effectively inhibit RNP activity.

### 3.3. Verification of Reactivation in the Presence of miRNA

**Figure 4:**
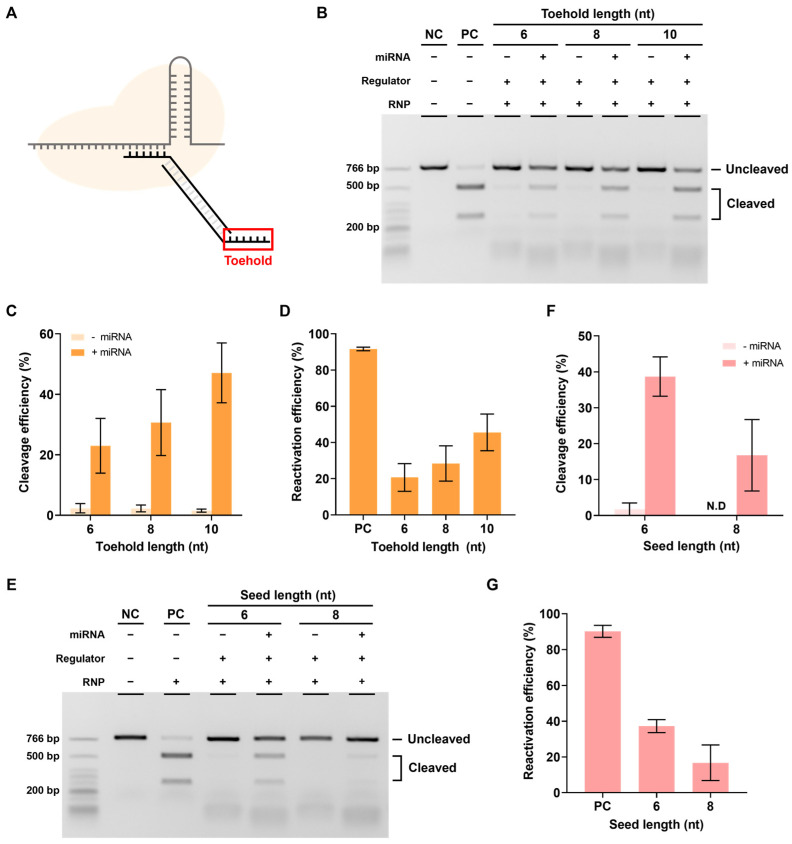
Reactivation efficiency as toehold length. (**A**) The toehold is the most important part of the reactivation of the RNP-regulator complex by miRNA. (**B**) The in vitro reactivation assay results determine the reactivation efficiency according to the presence or absence of miRNA using the seed 6 nt regulator with various toehold lengths. The RNP:regulator:miRNA molar ratio is 1:1:2 (**C**) Quantification of the ratio of cleaved target DNA from (**B**). (**D**) Quantification of the reactivation efficiency from (**B**). This reactivation efficiency was calculated as cleavage efficiency with miRNA–cleavage efficiency without miRNA). (**E**) In vitro reactivation assay results indicating the reactivation efficiency according to the presence or absence of miRNA using the toehold 10 nt regulator with various seed lengths. The RNP:Regulator:miRNA molar ratio is 1:1:2 (**F**). Quantification of the ratio of cleaved target DNA from (**E**). ND: not detected. (**G**) Quantification of the reactivation efficiency from (**E**). Error bars represent the mean ± SD from three independent experiments.

The next step involved performing experiments to determine whether reactivation was possible in the presence of miRNA. To this end, miR21, which is highly expressed in several cancers, was selected as the target miRNA [26,27,28,29], and the seed 6 nt regulator used earlier was introduced to verify the reactivation efficiency according to the toehold (TH) length (Figure 4A).

Reactivation efficiency was confirmed by measuring the cleavage efficiency when miR21 was added, which reactivated the RNP in a state where the DNA regulator inhibited its activity. As shown in Figure 4B,C, the cleavage efficiency was confirmed to be increased when miRNA was present as the length of TH increased. The actual reactivation efficiency was calculated as [cleavage efficiency when miRNA was present-cleavage efficiency when miRNA was absent] to exclude the background, and the reactivation efficiency was found to increase proportionally with the TH length (Figure 4D). These results showed that the RNP-regulator complex with inhibited activity can be properly reactivated by miRNAs.

However, if the binding between the seed and spacer is too strong, reactivation efficiency may decrease, even with a sufficiently long TH. Therefore, the effect of seed length on reactivation was also investigated. When the length of the seed was increased while keeping the length of the TH fixed at 10 nt, a decrease in target cleavage efficiency and reactivation efficiency was observed (Figure 4E–G). In other words, both the length of the TH and the length of the seed can affect reactivation and reactivation efficiency was found to be better with longer TH and shorter seed sequences.

After confirming that miRNA could reactivate the RNP-regulator complex, we investigated whether this phenomenon was due to the collapse of the DNA regulator structure caused by miRNA. We first confirmed whether strand displacement occurred through the TH of strand 2 when the regulator and miRNA were added together. By adding the regulator and miRNA together under the same conditions as in the in vitro cleavage assay, we observed the appearance of a new strand2 + miR21 band (3 band) (Supplementary Figure S2a). This result shows that miRNA can bind to the TH of the regulator and that strand displacement can occur along the stem.

Next, we investigated whether the regulator could detach when an RNP-regulator complex was formed (Supplementary Figure S2b). The sample with miRNA added to the RNP-regulator complex showed the presence of miRNA (3 band), miRNA + strand2 (2 bands), and some strand 1 (4 band) that had fallen off. This result indicates that even in the state where the RNP-regulator complex is formed, miRNA can bind to the TH of strand 2 and cause strand displacement, resulting in the collapse of the structure and falling off from the RNP. In other words, the results confirmed that the DNA regulator functioned as expected. However, the remaining RNP-regulator complex (red box) still indicated that not all regulators fall off because of miRNAs, which may be related to the reactivation efficiency being lower than that in the absence of a regulator.

### 3.4. Improvement of Reactivation Efficiency Using Locked Nucleic Acid (LNA)-Modified DNA Regulator

**Figure 5:**
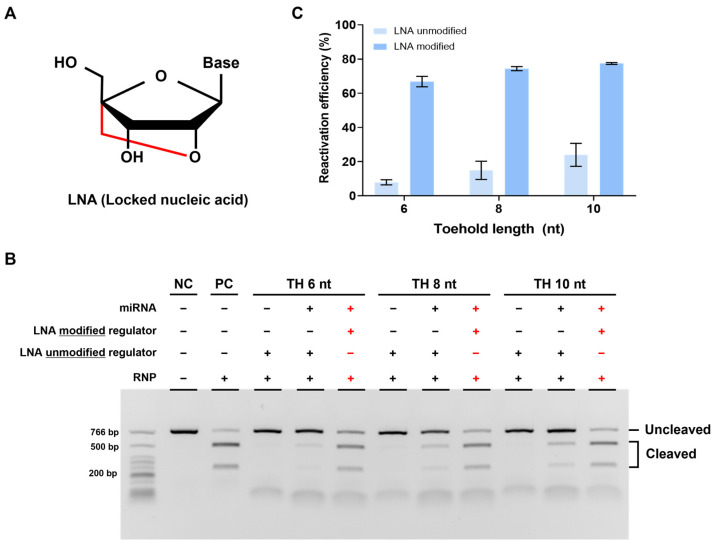
Enhanced reactivation efficiency by LNA modification. (**A**) Structure of LNA (locked nucleic acid) modified from the RNA monomer. It has a locked confirmation and improves binding efficiency between nucleic acids. (**B**) Results of in vitro cleavage analysis on the LNA-modified DNA regulator. The RNP:regulator:miRNA molar ratio is 1:1:2 (**C**). Quantification of the ratio of cleaved target DNA from (**B**). Error bars represent the mean ± SD from three independent experiments.

We confirmed the feasibility of our strategy but still observed a lower cleavage efficiency compared to that in cases without a regulator when reactivated using miRNA. We attributed this to the weak binding affinity between TH and miRNA and thus optimized it by introducing an LNA to TH. LNA is an RNA analog with a fixed (locked) structure that connects the 2′ oxygen and 4′ carbon of the ribose ring, which is known to increase binding affinity with complementary DNA (Figure 5A) [30].

To verify the effect of LNA, we designed a new strand 2 by modifying three nucleotides in TH with LNA. We then conducted experiments using the regulator, following the same procedure as before. As expected, reactivation efficiency was improved with the LNA-modified regulator compared to that with the unmodified regulator, indicating that LNA can increase the binding affinity between miRNA and regulator TH. Furthermore, when we applied the LNA modification to a regulator with a different TH length, we observed a greater improvement in the reactivation efficiency with the LNA-modified regulator (Figure 5B,C). In summary, the introduction of LNA into TH enhanced the binding affinity between miRNA and regulator TH, leading to increased reactivation efficiency.

### 3.5. Application to Different Targets

Based on a series of experiments, we found that using regulators modified with LNA in the toehold region yielded the highest reactivation efficiency. This optimal design was subsequently selected for further investigation. Therefore, we verified whether similar outcomes would be observed when applying DNA regulators to different target miRNAs. The miRNAs chosen for examination were let7a and miR221, both of which are relevant to cancer [31,32,33,34].

**Figure 6:**
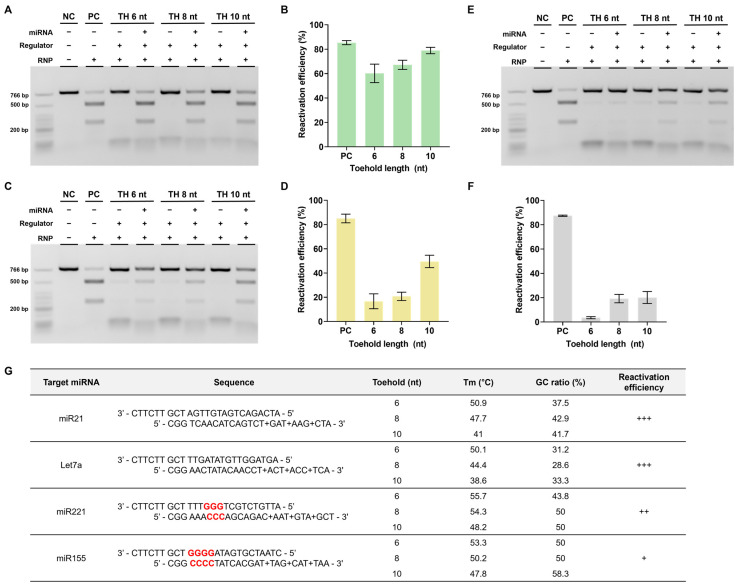
Confirmation of reactivation efficiency for different target miRNAs (**A**,**C**,**E**). Agarose gel result of the in vitro reactivation assay using LNA-modified regulator for let 7a and miR221. The RNP:regulator:miRNA molar ratio is 1:1:2 (**B**,**D**,**F**). Quantification of the ratio of cleaved target DNA from (**A**,**C**). (**G**) Table presenting the sequences of DNA regulator, GC content, and Tm in the regulator stem sequence as well as the reactivation efficiency for each target. +: low, ++: medium, +++: high.

To conduct these experiments, we modified the stem and toehold sequences of the regulator according to the altered sequences of the target miRNAs. In the case of let7a, the reactivation efficiency was found to be comparably high, similar to that of miR21 (Figure 6A,B). However, for miR221, the reactivation efficiency was notably lower than that of the previous two miRNAs (Figure 6C,D). We hypothesized that this difference could be attributed to variations in the GC content ratio within the stem of the DNA regulator. 

Considering the toehold-mediated strand displacement mechanism of the DNA regulator, a higher GC content ratio in the overall stem can increase the stability between the two DNA regulator strands, resulting in an elevated melting temperature (Tm). Furthermore, a concentrated GC ratio near the PAM within the stem, which is the crucial final region for completing strand displacement, can also contribute to stability, as mentioned earlier. This increased stability weakens miRNA-triggered strand displacement, potentially hindering this process. Consequently, if this process does not proceed smoothly, the reactivation efficiency is likely to decrease.

To validate this hypothesis, we conducted additional experiments using miR155, which has a high GC content ratio in the stem and adjacent PAM. In this case, despite a sufficient toehold length, the reactivation efficiency reached only approximately 20% (Figure 6E,F). These findings emphasize the importance of avoiding a high GC content ratio when designing DNA regulators (Figure 6G).

## 4. Discussion

This study introduced a novel and design-friendly method for regulating CRISPR activity using a DNA regulator that responds to miRNA. This approach, simpler than modifying proteins or gRNA, effectively demonstrated the activation and inhibition of the Cas9 RNP complex in response to specific miRNAs. Moreover, the introduction of the LNA into the toehold region maximized reactivation efficiency; thus, we proposed guidelines for an optimized DNA regulator design based on these results.

While other studies have also developed miRNA-responsive CRISPR systems similar to ours, they have generally integrated miRNA-responsive regions directly into Cas9 or gRNA or designed complex structures responsive to miRNA [8,11,12,35]. Although these methods can achieve specificity, they require complex synthetic processes for the modified components and additional steps to create the necessary DNA structures. In contrast, our method employs a DNA regulator that easily targets various genes with simple sequence changes and functions by simply mixing with RNP. Consequently, the proposed approach offers a simple design. 

**Figure 7:**
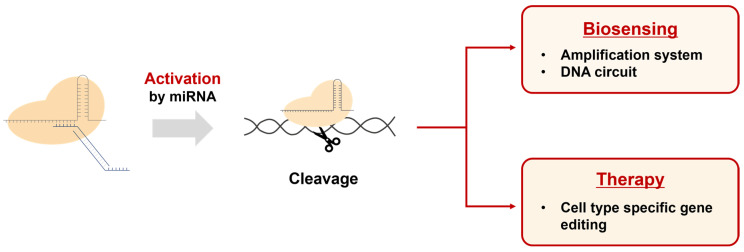
Applications of the DNA regulator-based CRISPR activation system. This system can be used in the field of biosensing for the detection of miRNAs by connecting them with amplification and DNA circuits. Additionally, the CRISPR system activated by miRNA can be used to edit genes in specific cell types for therapeutic purposes.

Our results demonstrate that this system can precisely control gene editing processes in the presence of specific cellular conditions or signals. These characteristics enable our DNA regulator-based CRISPR activation system to be utilized in various potential applications (Figure 7). First, it can be used to develop biosensors capable of detecting specific miRNAs. By activating the CRISPR system with miRNA and subsequently combining it with amplification systems or other genetic circuits, innovative biosensing platforms can be created. Second, it allows the CRISPR system to be utilized as a cell-specific therapeutic agent when introduced into cellular systems. As miRNA is known to be expressed in a cell type-specific manner, our approach can enable CRISPR-mediated gene editing only in specific cells. Furthermore, we believe that our system can be extended to various CRISPR variants, such as dCas9 and base editors, enhancing its potential as a flexible genome editing tool.

However, some potential limitations need to be considered. The efficiency and specificity of miRNA recognition and response may vary between cell and tube conditions, necessitating further validation and optimization. Additionally, the dynamic nature of miRNA expression can impact the performance of our system in certain contexts. 

Therefore, future studies should focus on exploring the applications of different biosensing platforms, validating their performance in cellular models, and addressing the potential challenges related to specificity and efficiency. This effort will ensure that this novel method for regulating CRISPR activity and its applications can be effectively utilized in the rapidly evolving fields of biosensors, genome editing, and gene therapy. 

Overall, we successfully demonstrated the modulation of CRISPR activity by miRNAs in vitro. The DNA regulator is biocompatible and can target multiple genes with a simple sequence change, thus proving design-friendly and highly versatile. We anticipate that this method will effectively control CRISPR and can be used in various applications, including therapeutics and biosensing.

## Data Availability

Not applicable.

## Supplementary Materials

The following supporting information can be downloaded at: https://www.mdpi.com/article/10.3390/bios13110975/s1, Figure S1: Confirmation of inhibitory effect by single-stranded DNA regulator; Figure S2: Validation of miRNA response of the DNA regulator; Table S1: Summary of oligonucleotide sequences.
