# Supplementary material for: MiRNA-Responsive CRISPR-Cas System via a DNA Regulator
Source: Biosensors (Basel). 2023 Nov 7;13(11):975. doi: 10.3390/bios13110975 (PMC10669420; doi:10.3390/bios13110975)
Supplement: Supplementary file 1 [file biosensors-13-00975-s001.zip › biosensors-2645168-supplementary.pdf]

Supporting Information for

MiRNA-responsive CRISPR-Cas system via a DNA regulator

Da young Yun, Cheulhee Jung *

*Corresponding Author: Cheulhee Jung

Email: damo363@korea.ac.kr

This file includes:

Supplementary Figure S1: Confirmation of inhibitory effect by single strand DNA regulator.

Supplementary Figure S2: Validation of miRNA-response of the DNA regulator.

Supplementary Table S1: Summary of oligonucleotides sequence.

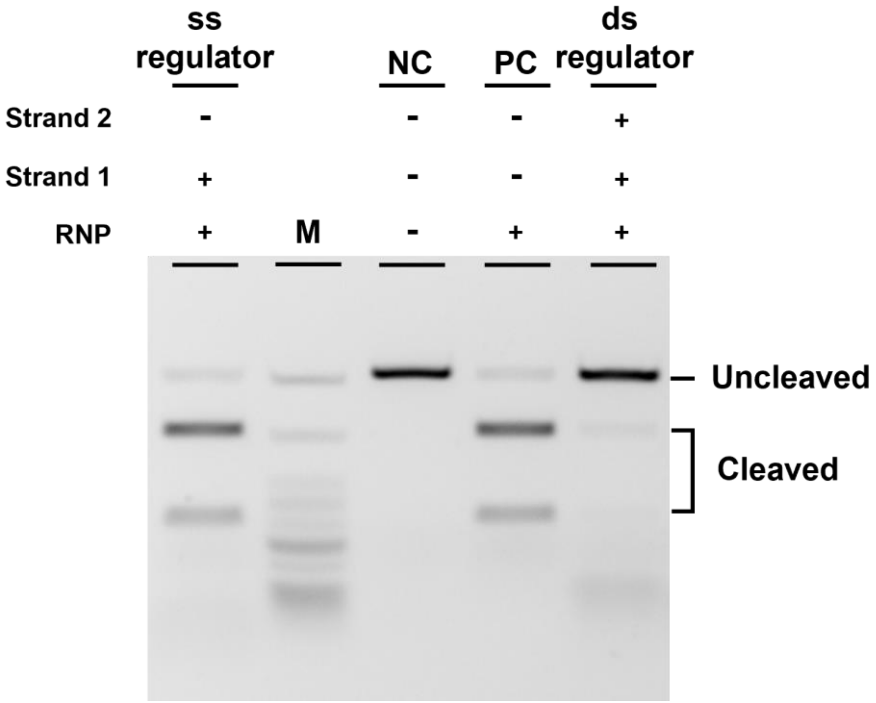

Figure S1. Confirmation of inhibitory effect by single strand DNA regulator. In vitro cleavage assay result to prove the effect of single strand(ss) regulator. ss regulator has similar cleavage efficiency with PC. It means that the single strand regulator cannot inhibit the RNP activity. M; Marker.

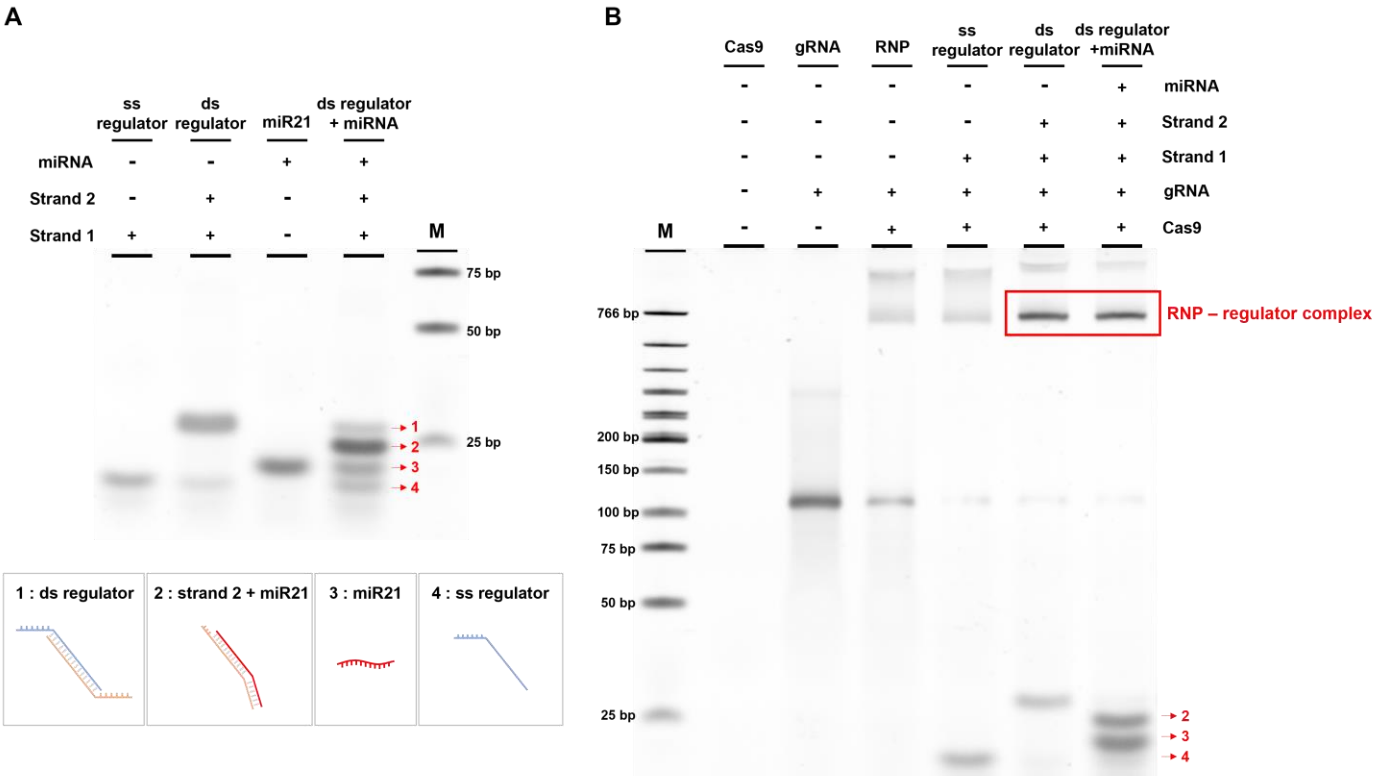

Figure S2. Validation of miRNA-response of the DNA regulator using native PAGE gel. (**A**) Identification of toehold displacement of the DNA regulator by miR21. When miR21 is present, it binds with TH of strand 2 and then strand displacement occurs along the stem. Eventually, the newly appeared strand 2 + miR21 band emerged. This result means that the regulator structure was successfully disrupted by miR21. (**B**) EMSA assay result for the RNP-regulator complex. The RNP-regulator complex is formed only when the double strand regulator is present. Also, the strand 2 + miR21 band indicates that the regulator can be released from the complex when miR21 is added. M; Marker

Table S1. Summary of oligonucleotides sequence

LNA modification is indicated by +.

**sgRNA and primer**	
EMX1 sgRNA	GAGTCCGAGCAGAAGAAGAA
EMX1 Forward primer	GGGTCATAGGCTCTCTCATTTAC
EMX1 Reverse primer	CCATTGCTTGTCCCTCTGT
**miR21 related oligo**	
miRNA21	UAGCUUAUCAGACUGAUGUUGA
miR21_1-1_S4_TH6	ATCAGACTGATGTTGA TCG TTCT
miR21_1-1_S5_TH6	ATCAGACTGATGTTGA TCG TTCTT
miR21_1-1_S6_TH6	ATCAGACTGATGTTGA TCG TTCTTC
miR21_1-1_S6_TH8	CAGACTGATGTTGA TCG TTCTTC
miR21_1-1_S6_TH10	GACTGATGTTGA TCG TTCTTC
miR21_1-1_S8_TH6	ATCAGACTGATGTTGA TCG TTCTTCTT
miR21_1-1_S8_TH10	GACTGATGTTGA TCG TTCTTCTT
miR21_1-2	CGG TCAACATCAGTCTGATAAGCTA
miR21_1-2_TH LNA	CGG TCAACATCAGTCT+GAT+AAG+CTA
**let 7a related oligo**	
let 7a	UGAGGUAGUAGGUUGUAUAGUU
let 7a_1-1_S6_TH6	AGTAGGTTGTATAGTT TCG TTCTTC
let 7a _1-1_S6_TH8	TAGGTTGTATAGTT TCG TTCTTC
let 7a _1-1_S6_TH10	GGTTGTATAGTT TCG TTCTTC
let 7a_1-2_TH LNA	CGG AACTATACAACCT+ACT+ACC+TCA
**miR221 related oligo**	
miR221	AGCUACAUUGUCUGCUGGGUUUC
miR221_1-1_S6_TH6	ATTGTCTGCTGGGTTT TCG TTCTTC
miR221_1-1_S6_TH8	TGTCTGCTGGGTTT TCG TTCTTC
miR221_1-1_S6_TH10	TCTGCTGGGTTT TCG TTCTTC
miR221_1-2_TH LNA	CGG AAACCCAGCAGAC+AAT+GTA+GCT

**miR155 related oligo**	
miR155	UUAAUGCUAAUCGUGAUAGGGGUU
miR155_1-1_S6_TH6	CTAATCGTGATAGGGG TCG TTCTTC
miR155_1-1_S6_TH8	AATCGTGATAGGGG TCG TTCTTC
miR155_1-1_S6_TH10	TCGTGATAGGGG TCG TTCTTC
miR155_1-2_TH LNA	CGG CCCCTATCACGAT+TAG+CAT+TAA
